# Phosphorylated vasodilator-stimulated phosphoprotein (P-VASP^Ser239^) in platelets is increased by nitrite and partially deoxygenated erythrocytes

**DOI:** 10.1371/journal.pone.0193747

**Published:** 2018-03-05

**Authors:** Sirada Srihirun, Barbora Piknova, Nathawut Sibmooh, Alan N. Schechter

**Affiliations:** 1 Molecular Medicine Branch, National Institute of Diabetes Digestive and Kidney Diseases, National Institutes of Health, Bethesda, Maryland, United States of America; 2 Department of Pharmacology, Faculty of Dentistry, Mahidol University, Bangkok, Thailand; 3 Department of Pharmacology, Faculty of Science, Mahidol University, Bangkok, Thailand; Albany Medical College, UNITED STATES

## Abstract

Nitrite is recognized as a bioactive nitric oxide (NO) metabolite. We have shown that nitrite inhibits platelet activation and increases platelet cGMP levels in the presence of partially deoxygenated erythrocytes. In this study, we investigated the effect of nitrite on phosphorylation of vasodilator-stimulated phosphoprotein on residue serine 239 (P-VASP^Ser239^), a marker of protein kinase G (PKG) activation, in human platelets. In platelet-rich plasma (PRP), nitrite itself had no effect on levels of P-VASP^Ser239^ while DEANONOate increased P-VASP^Ser239^. Deoxygenation of PRP + erythrocytes (20% hematocrit) raised baseline P-VASP^Ser239^ in platelets. At 20% hematocrit, nitrite (10 μM) increased P-VASP^Ser239^ in platelets about 31% at 10–20 minutes of incubation while the levels of P-VASP^Ser157^, a marker of protein kinase A (PKA) activation, were not changed. Nitrite increased P-VASP^Ser239^ in platelets in the presence of deoxygenated erythrocytes at 20–40% hematocrit, but the effects were slightly greater at 20% hematocrit. In conclusion, our data confirm that nitrite increases P-VASP^Ser239^ in platelets in the presence of deoxygenated erythrocytes. They also further support the idea that partially deoxygenated erythrocytes may modulate platelet activity, at least in part, via the NO/sGC/PKG pathway from NO formed by reduction of circulating nitrite ions.

## Introduction

Nitrite is now recognized as a bioactive NO metabolite that can be converted to NO either by enzymatic reaction or non-enzymatic disproportionation under hypoxia and low pH [1, 2] The vasodilatory effect of nitrite has been extensively studied during the last decade; however the effect of nitrite on platelets received little attention until the last several years. Publications from our group starting in 2012 showed that while nitrite itself at physiological concentrations had little effect on platelets, interaction between partially deoxygenated erythrocytes with nitrite inhibited platelet aggregation by the release of NO [3]. The effects were seen in human and rodent platelets with several different agonists [4, 5]. The effect of nitrite on platelets has been confirmed by aggregometry, flow cytometry and thromboelastography by several independent laboratories as well as our own group [6–10]. Since nitrite in the presence of erythrocytes increased platelet cGMP levels, we suggested that nitrite inhibited platelet activation through NO/sGC pathway [3, 6]. However, the effect of nitrite on P-VASP^Ser239^, one marker of sGC/PKG activation in platelets has been questioned. A recent publication failed to detect the effect of nitrite and deoxygenated erythrocytes on P-VASP^Ser239^ levels in platelets [11]. These issues are important both because of the potential relevance of vascular erythrocytes to the blood clotting process and its pharmacology but also because changes in this pathway have been interpreted as the most definitive evidence for the ability of erythrocytes to generate NO from nitrite by reductive chemical processes.

Phosphorylation of VASP, an actin binding protein, inhibits activation of glycoprotein IIb/IIIa [12, 13]. Although PKG phosphorylates several signaling proteins in platelets, VASP, which is highly expressed in platelets, is considered as an important marker of sGC/cGMP/PKG activation [14, 15]. VASP phosphorylation at serine 239 is specific for PKG activation while phosphorylation at serine 157 is responsible for PKA pathway [16]. In contrast to a publication from Gambaryan et. al. [11], our recent data showed increased P-VASP^Ser239^ in human platelets *in vitro* by nitrite in deoxygenated whole blood and *ex vivo* after nitrite inhalation [17]. We have extended our previous study to investigate the role of deoxygenation and hematocrit on P-VASP^Ser239^ induced by nitrite ions in human platelets. The effects of nitrite and NO donors on platelet P-VASP^Ser239^ in whole blood were measured under various conditions to better understand this phenomenon.

## Materials and methods

### Subjects

Blood samples were obtained from healthy donors on the IRB approved National Institutes of Health protocol 99-CC-0168. Research blood donors were randomly recruited by the Department of Transfusion Medicine since December 2016 to December 2017 by phone, email or flyers at NIH from the local community. Donors were non-pregnant adults who met healthy blood donor criteria and tested negative for transfusion-transmissible diseases. The written informed consent was obtained in accordance with the declaration of Helsinki. Blood samples were collected in acid citrate dextrose (ACD) and used within 3 hours of withdrawal.

### Sample preparation

Whole blood was centrifuged at 120×g for 10 minutes at room temperature to obtain PRP. Erythrocytes were obtained from centrifugation of the lower portion at 2500×g for 10 minutes. Whole blood, PRP and PRP plus erythrocytes added to various hematocrit levels were used in further experiments.

Nitrite (10 μM) or DEANONOate (1 μM) was incubated in PRP at 37°C for 5, 10, 15, 20, 40 and 60 minutes. Then, platelets were separated by centrifugation and collected in lysis buffer (50 mM Tris, 0.5% NP-40 and 150 mM NaCl) with a proteinase inhibitor cocktail III (1:500, Calbiochem La Jolla, CA).

Under oxygen controlled experiments, whole blood or PRP + erythrocytes (10, 15, 20, 30 and 40% hematocrit) were deoxygenated by gentle blowing helium above cell suspension for 10 minutes with a final partial oxygen pressure (pO_2_) = 24.67 ± 1.45 mmHg. The pO_2_ was measured by ABL-80 FLEX CO-OX blood gas analyzer (Radiometer American Inc.). Levels of cell-free hemoglobin before and after deoxygenation were measured by a human hemoglobin ELISA kit (Abcam, Cambridge, MA). Nitrite 10 μM was injected into the preparation and incubated for 5, 10, 15, 20, 30, 40 and 60 minutes at 37°C. In another set of experiment, 300 μM L-NAME or 10 μM ODQ was incubated with PRP + erythrocytes (20% hematocrit) for 30 and 10 minutes, respectively. Platelets were collected after 10-minute incubation with 10 μM nitrite in deoxygenated samples.

In a separate set of experiments, DEANONOate (1 μM) and sodium nitroprusside (10 μM) were incubated in PRP, whole blood and deoxygenated whole blood for 5 minutes at 37 °C. Since our preliminary data in deoxygenated whole blood showed that nitrite maximally increased P-VASP^Ser239^ at 15-minute of incubation, nitrite was added to deoxygenated whole blood for 15 minutes. Platelets were collected in lysis buffer plus proteinase inhibitors.

### Western blots

Protein (15 μg) extracts from the platelets were separated by 10% SDS-PAGE and transferred to nitrocellulose membranes. Membranes were blocked with 2% non-fat dry milk for 1 hour and incubated with anti-VASP^Ser239^ (Cat. no. 3114), anti-VASP^Ser157^(Cat. no. 3111), anti-VASP (Cat. no. 3112) and GAPDH (Cat. no. 2118) overnight (Cell Signaling Technology, Danvers MA). Goat anti-rabbit conjugated with horseradish peroxidase (Jackson ImmunoResearch, West Grove, PA) was used as a secondary antibody and followed by ECL detection (Bio-Rad, Hercules, CA). Band density was qualified by NIH ImageJ software.

### Statistics

All data are presented as means ± SEM. Data processing and statistical analysis were done by GraphPad Prism^®^ Version 4 (GraphPad software Inc., San Diego, CA).

## Results

### Nitrite ions alone had little effect on P-VASP^Ser239^ levels in platelets

**Fig 1:**
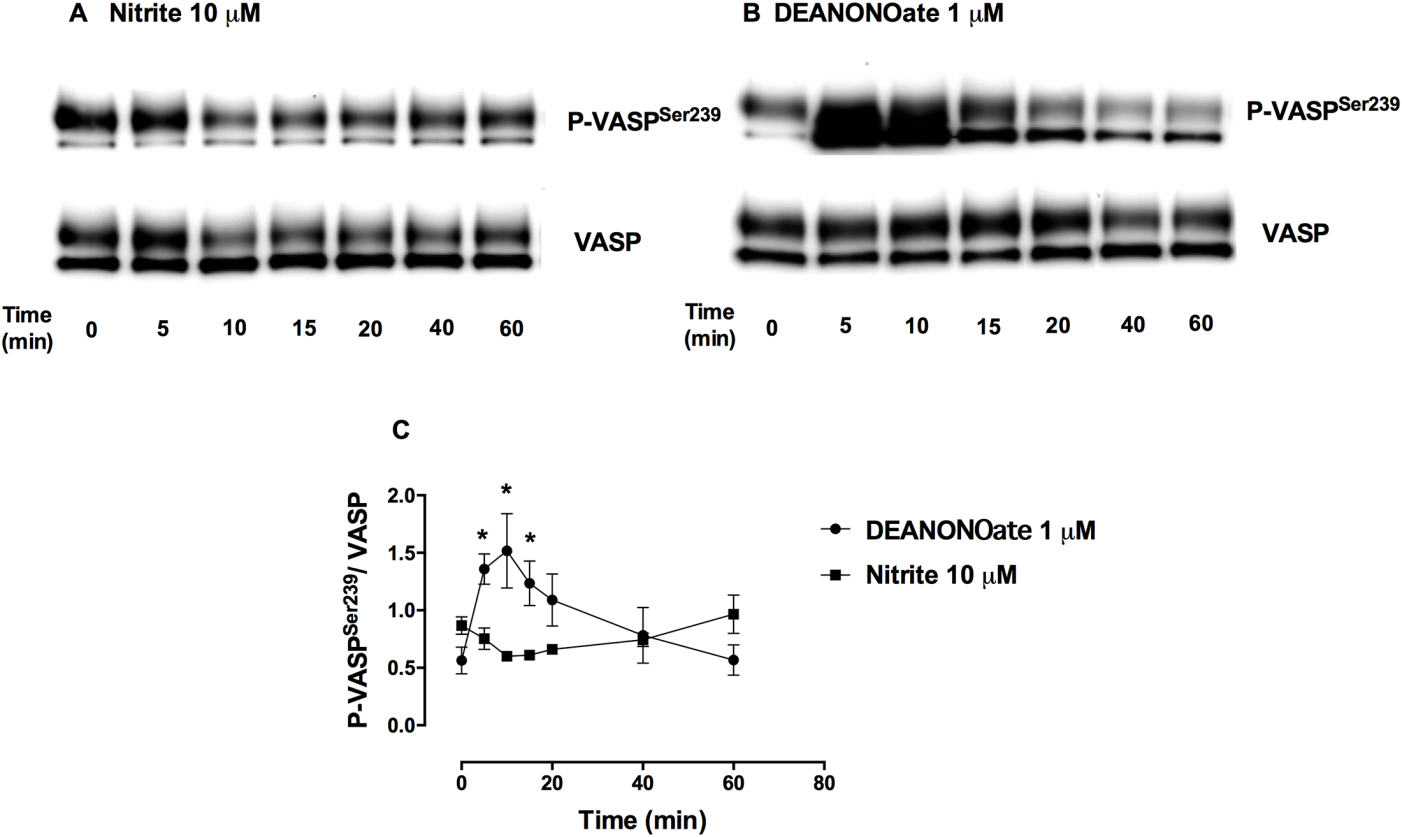
Increased P-VASP^Ser239^ induced by DEANONOate but not nitrite in PRP. Representative Western blot bands of P-VASP^Ser239^ and VASP expression in PRP treated with 10 μM nitrite (A) or 1 μM DEANONOate (B) and quantification of P-VASP^Ser239^/VASP (C) at 37 °C for 5, 10, 15, 20, 40 and 60 minutes are shown. Data are mean ± SEM (n = 3). *P < 0.05 tested by Student’s t-test.

Nitrite (10 μM) incubated in PRP for 5 to 60 minutes had small effects on P-VASP^Ser239^ levels in platelets while DEANONOate (1 μM) significantly increased P-VASP^Ser239^ at 5 minutes after incubation. The increased P-VASP^Ser239^ induced by DEANONOate reached a maximum at 10-minute of incubation and returned to baseline at 40–60 minutes (Fig 1).

### Nitrite + erythrocytes increased P-VASP^Ser239^ levels in platelets under deoxygenation

**Fig 2:**
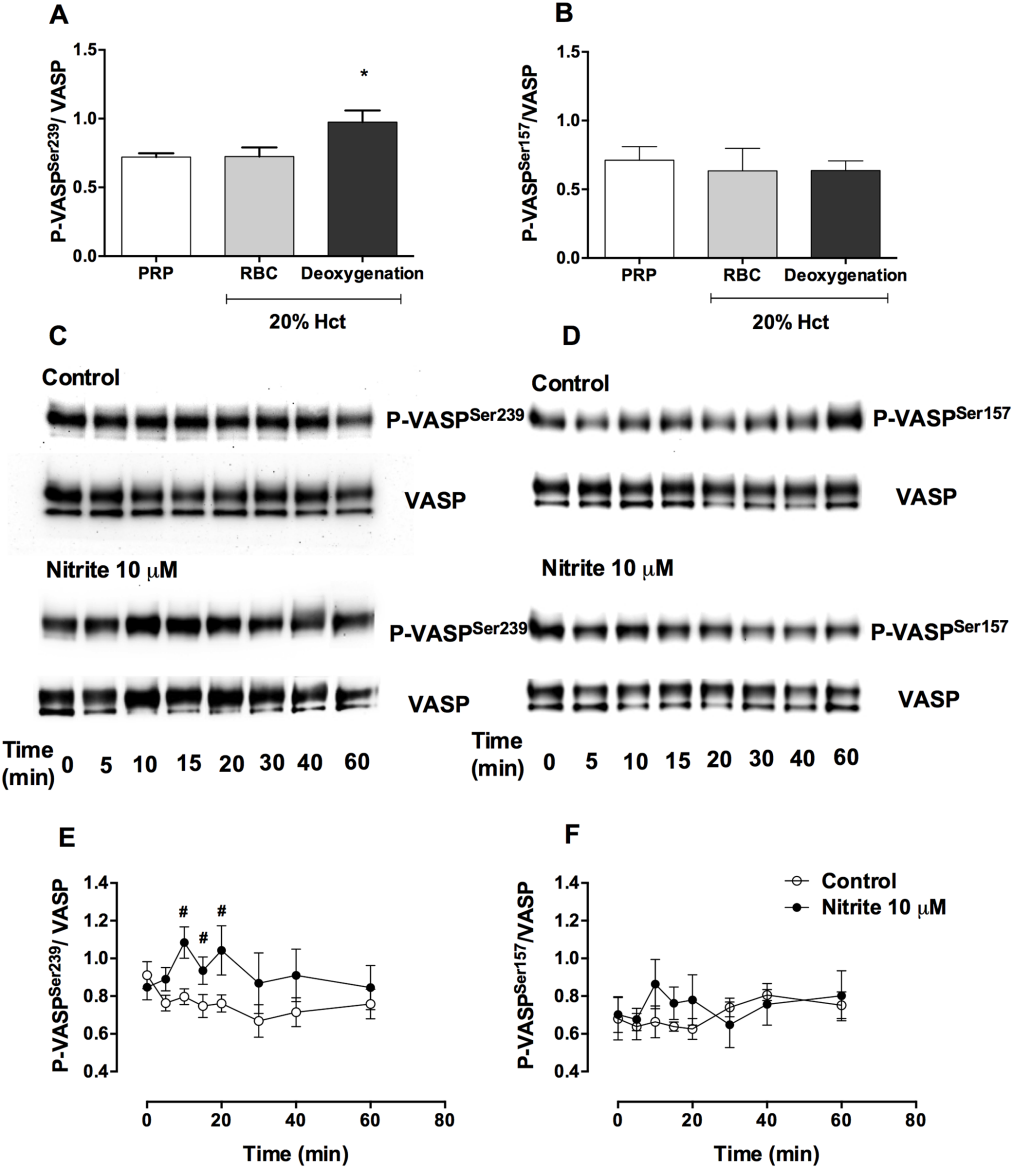
Nitrite increased P-VASP^Ser239^ but not P-VASP^Ser157^ in the presence of deoxygenated erythrocytes. Quantification of baseline P-VASP^Ser239^/ VASP (A) and P-VASP^Ser157^/ VASP (B) in platelets from PRP, PRP + erythrocytes (20% hematocrit) with or without deoxygenation, representative Western blot bands of P-VASP^Ser239^ (C), P-VASP^Ser157^ (D), VASP expression and quantification of P-VASP^Ser239^/VASP (E) and P-VASP^Ser157^/VASP (F) in control and nitrite treated deoxygenated PRP + erythrocytes (20% hematocrit) at different time points are shown in this figure. PRP + erythrocytes (20% hematocrit) were deoxygenated by helium for 10 minutes (pO_2_ ~ 25 mmHg). Nitrite (10 μM) was added in deoxygenated samples and incubated for 5, 10, 15, 20, 30, 40 and 60 minutes. Data are mean ± SEM (n = 7). *P < 0.05 compared with PRP and tested by one-way ANOVA with Tukey’s multiple comparison. ^#^*P* < 0.05 compared with control and tested by paired Student’s t-test.

Because we previously found that effect of nitrite on platelet aggregation in the presence of erythrocytes was maximal at about 20% hematocrit, PRP and erythrocytes at 20% hematocrit were used in these experiments. After deoxygenation (pO_2_ ~ 25 mmHg), baseline P-VASP^Ser239^ but not P-VASP^Ser157^ increased in platelets (Fig 2A and 2B). A 10-minute incubation of nitrite (10 μM) increased P-VASP^Ser239^ in platelets by 31.15 ± 5.78% but levels did not increase without deoxygenation. Addition of nitrite raised P-VASP^Ser239^ at 10 to 20 minutes and values returned to baseline within 1 hour (Fig 2C and 2E). In addition, nitrite at 1 μM increased P-VASP^Ser239^ by 18.87 ± 5.62% at 10-minute incubation (data not shown). In contrast, addition of nitrite at this concentration, with or without erythrocytes did not alter P-VASP^Ser157^ in platelets (Fig 2D and 2F). L-NAME, a nitric oxide synthase (NOS) inhibitor, and ODQ, a soluble cyclase inhibitor, both decreased baseline P-VASP^Ser239^ associated with the deoxygenation of PRP + erythrocytes. In addition, both L-NAME and ODQ, inhibited P-VASP^Ser239^ induced by exogenous nitrite (S1 Fig). These results suggest that NOS either from the erythrocytes or the platelets themselves plays some in nitrite conversion to NO by erythrocytes. However, further studies are required to evaluate this mechanism.

**Fig 3:**
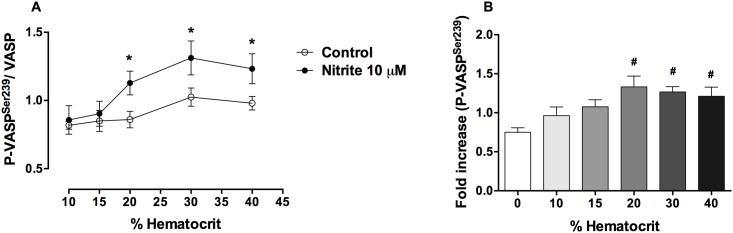
Nitrite effects on P-VASP^Ser239^ in platelets in the presence of deoxygenated erythrocytes at different hematocrit values. Quantification of P-VASP^Ser239^/VASP (A) and fold increase (B) of P-VASP^Ser239^ expression in control and nitrite treated platelets at different hematocrit values are shown. PRP alone or PRP + erythrocytes (10, 15, 20, 30 and 40% hematocrit) were deoxygenated by helium. Nitrite (10 μM) was added to deoxygenated samples and incubated at 37 °C for 10 minutes. Data are mean ± SEM (n = 5). *P < 0.05 compared with control and tested by paired Student’s t-test. ^#^*P* < 0.05 compared with 0% hematocrit and tested by one-way ANOVA with Tukey’s multiple.

In order to investigate the influence of hematocrit on the nitrite effect, deoxygenated PRP + erythrocytes at 10, 15, 20, 30 and 40% hematocrit were incubated with 10 μM nitrite for 10 minutes. Cell-free hemoglobin levels at baseline (before deoxygenation) were 2.9 ± 0.8 μM. After deoxygenation, cell-free hemoglobin at 10, 15, 20, 30 and 40% hematocrit were 3.1 ± 0.7, 4.2 ± 0.4, 4.0 ± 0.5, 5.0 ± 0.5 and 5.3 ± 0.2 μM, respectively. Deoxygenation of samples with increasing hematocrit values increased cell-free hemoglobin levels slightly, but this effect was not statistically significant. At 10% and 15% hematocrit, nitrite did not increase P-VASP^Ser239^. However, nitrite increased P-VASP^Ser239^ in platelets at hematocrit values about 20% and above with the maximal effect at about 20% hematocrit (Fig 3, S2 Fig).

### Erythrocytes diminished P-VASP^Ser239^ expression induced by NO donors

**Fig 4:**
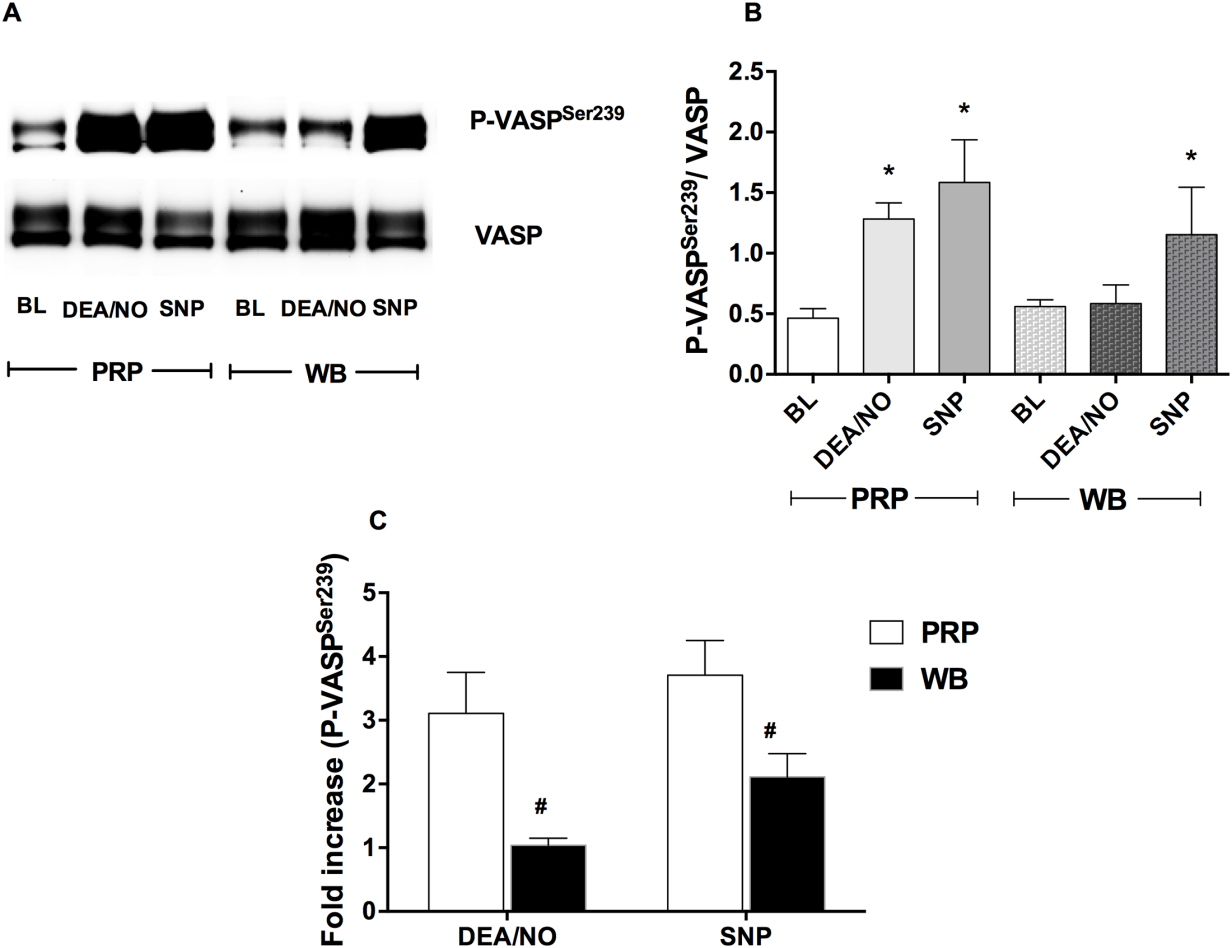
Erythrocytes diminished P-VASP^Ser239^ induced by NO donors. Representative Western blot bands of P-VASP^Ser239^ and VASP expression in platelets (A) and quantification of P-VASP^Ser239^/VASP (B) and fold increase of P-VASP^Ser239^ expression (C) in platelets in PRP and whole blood treated with DEANONOate and sodium nitroprusside are shown. DEANONOate (1 μM) and sodium nitroprusside (10 μM) were incubated in PRP or venous whole blood (WB) at 37 °C for 5 minutes. Data are mean ± SEM (n = 4). *P < 0.05 compared with baseline (BL) and tested by one-way ANOVA with Tukey’s multiple comparison. ^#^*P* < 0.05 compared with PRP and tested by paired Student’s t-test. DEA/NO = DEANONOate, SNP = Sodium nitroprusside.

Although NO donors dramatically increased P-VASP^Ser239^ in PRP, this effect was decreased by addition of erythrocytes. In PRP, P-VASP^Ser239^ induced by DEANONOate (1 μM) and sodium nitroprusside (10 μM) increased by 3.19 ± 0.90 and 3.60 ± 0.76-fold, respectively. However, the effect of DEANONOate was abolished in whole blood while sodium nitroprusside maintained some effect on P-VASP^Ser239^ (Fig 4A and 4B). However, the P-VASP^Ser239^ induced by sodium nitroprusside in whole blood was significantly decreased compared to its effect in PRP (Fig 4C).

### Nitrite was as effective as sodium nitroprusside for induction of P-VASP^Ser239^ in platelets

**Fig 5:**
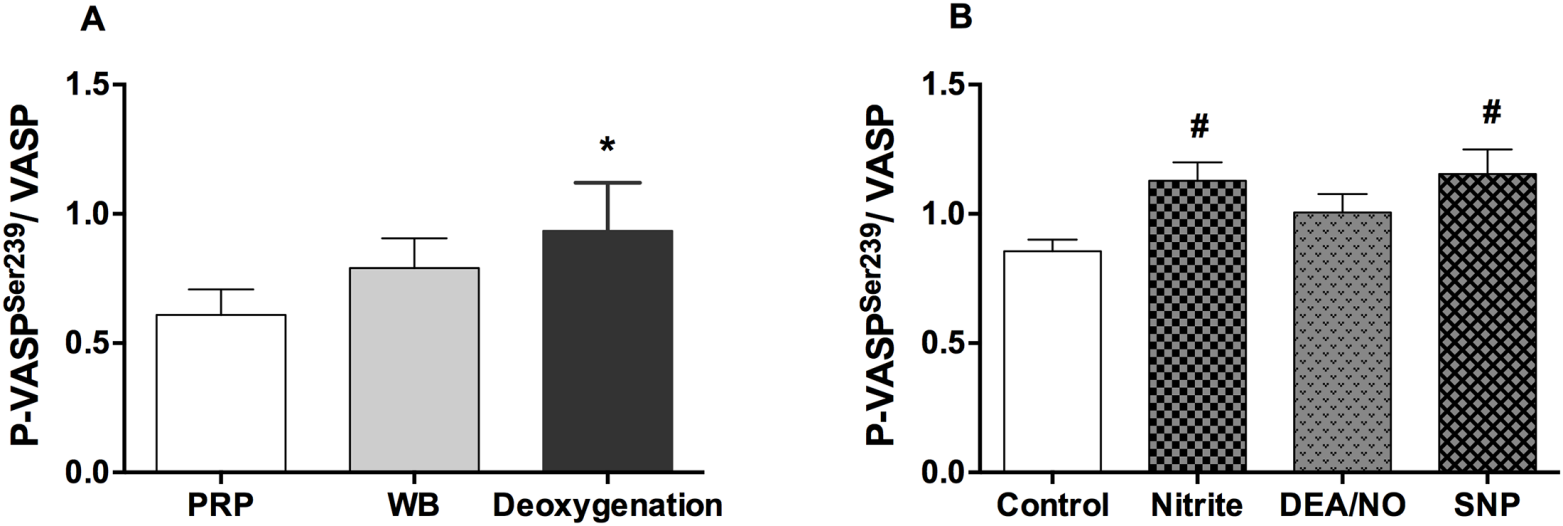
Comparative effects of nitrite and NO donors in deoxygenated whole blood. Quantification of P-VASP^Ser239^/VASP at baseline (A) and after treatment of deoxygenated whole blood with nitrite, DEANONOate and sodium nitroprusside (B) are shown. Whole blood was deoxygenated by helium for 10 minutes. Nitrite (10 μM) was incubated in deoxygenated whole blood for 15 minutes while DEANONOate (1 μM) and sodium nitroprusside (10 μM) were incubated for 5 minutes, respectively. Data are mean ± SEM (n = 5). *P < 0.05 compared with PRP and tested by one-way ANOVA with Tukey’s multiple comparison. ^#^*P* < 0.05 compared with control and tested by one-way ANOVA with Tukey’s multiple comparison.

In order to compare the effects of nitrite on P-VASP^Ser239^ with NO donors in platelets, levels of P-VASP^Ser239^ induced by nitrite (10 μM), DEANONOate (1 μM) and sodium nitroprusside (10 μM) were measured in deoxygenated whole blood. Without addition of nitrite, deoxygenated whole blood (pO_2_ ~ 25 mmHg) increased baseline P-VASP^Ser239^ in platelets slightly (Fig 5A). Nitrite and sodium nitroprusside significantly increased P-VASP^Ser239^ by 37.03 ± 5.11 and 32.99 ± 13.56%, respectively under these conditions while DEANONOate had little effect (Fig 5B, S3 Fig). In addition, DEANONOate at 10 μM did not increase P-VASP^Ser239^ in deoxygenated whole blood (data not shown). Therefore, nitrite is as effective as sodium nitroprusside in increasing P-VASP^Ser239^ in platelets in deoxygenated whole blood.

## Discussion

Our results demonstrated increased P-VASP^Ser239^ in platelets by nitrite in the presence of erythrocytes under deoxygenated condition while nitrite itself at 10 μM had no effect on PRP. Nitrite increased P-VASP^Ser239^ in platelets at 20% hematocrit and above. Under hypoxic condition, nitrite was as effective as sodium nitroprusside for inducing P-VASP^Ser239^ in platelets.

Our finding of increased P-VASP^Ser239^ in platelets induced by nitrite + erythrocytes corroborates the notion of platelet inhibition, at least in part, through the sGC/PKG pathway. In agreement with a previous study [18], nitrite alone did not have effect on P-VASP^Ser239^ in platelets. However, even without addition of exogenous nitrite, deoxygenation of PRP + erythrocytes raised baseline P-VASP^Ser239^. Decreasing pO_2_ with deoxygenation facilitates endogenous erythrocyte nitrite conversion to NO leading to increase P-VASP^Ser239^ in platelets. In order to study the kinetics of P-VASP^Ser239^ induced by nitrite, 10 μM nitrite (a possible pharmacological concentration of nitrite) was used in this study. In contrast to the rapid effect of DEANONOate, nitrite and deoxygenated erythrocytes increased P-VASP^Ser239^ after 10 to 20-minute of incubation; this increase returned to baseline at 60 minutes of incubation. Since nitrite conversion to NO requires complex processes including nitrite uptake, conversion to NO and export the NO out of the erythrocytes, the kinetics of PKG activation induced by nitrite and deoxygenated erythrocytes might be expected to be slower compared to the effect of spontaneous NO release from DEANONOate. Although NO has also been shown to inhibit Rap1b, a glycoprotein IIb/IIIa activator protein, via a VASP-independent pathway [19], phosphorylation of VASP via the sGC/PKG pathway appears to play an important role for the platelet inhibitory effect by nitrite and deoxygenated erythrocytes.

Since P-VASP^Ser157^ shifts the molecular weight of VASP from 46 kDa to 50 kDa [16], we tested whether nitrite increased P-VASP^Ser157^. We used the same set of samples to detect both Ser157 and Ser239 phosphorylation by Western blots and found that deoxygenation and addition of nitrite did not alter P-VASP^Ser157^ in platelets. This indicated that the effect of nitrite detected at 50-kDa-band is specific for the phosphorylation at serine 239.

Under deoxygenation, nitrite increased P-VASP^Ser239^ in platelets in the presence of erythrocytes at and above 20% hematocrit. We found that P-VASP^Ser239^ induced by nitrite reached maximum effect at about 20% hematocrit. Deoxygenation of PRP + erythrocytes induced insignificant increases in cell-free hemoglobin levels, therefore the slightly decreased P-VASP^Ser239^ induced by nitrite in the presence of erythrocytes at 30 and 40% hematocrit was not due to cell free hemoglobin from the deoxygenation process. These results are consistent with our previous platelet inhibitory effect of nitrite detected by both aggregometery and thromboelastography [3, 20]. We showed that nitrite + erythrocytes at 10 and 15% hematocrit had little effect on P-VASP^Ser239^ in platelets. This may explain why a recent publication failed to demonstrate effect of nitrite on P-VASP^Ser239^ in platelets in the presence of erythrocytes at 0.9% hematocrit (10^8^ RBCs/mL) [11]. The fact that nitrite was added to the erythrocytes before adding the platelets may also account for these negative results.

The decreased effect of NO donors on platelets by erythrocytes has been reported previously [21]. Our studies found that DEANONOate caused 3-fold increase in P-VASP^Ser239^ in PRP however; this effect was abolished by the presence of erythrocytes. At physiological pH (37 °C), NO is rapidly released (half-life = 2 minutes) from DEANONOate and taken up into erythrocytes. In erythrocytes, NO reacts with either oxyhemoglobin to form nitrate + methemoglobin or deoxyhemoglobin to form iron nitrosyl hemoglobin (HbNO) [22, 23]. This suggests that NO release spontaneously by DEANONOate and taken up by erythrocytes would not be able to escape erythrocytes. This may also explain why high hematocrit levels reduce the effects of nitrite.

In contrast, sodium nitroprusside could increase platelets P-VASP^Ser239^ in the presence of erythrocytes in venous and deoxygenated whole blood. A previous study showed that sodium nitroprusside inhibited platelet aggregation in both PRP and whole blood [24]. Although sodium nitroprusside is clinically used to decreased blood pressure in hypertensive emergency, the mechanism of NO release remains incompletely understood [25]. NO release from nitroprusside requires an electron transfer from sulfhydryl-containing compounds (glutathione and cysteine) and leads to formation of disulfides and S-nitrosothiols [26]. S-nitrosothiol is detected in tissue after addition of sodium nitroprusside and is believed to be a part of storage and transport of NO *in vivo* [27].

Nitrite reductase activity of hemoglobin reaches a maximum rate at 50% oxygen saturation (pO_2_ ~ 28 mmHg) [28, 29], which is close to values found in tissues such as muscle and skin [30]. This suggests that nitrite can be converted to NO under hypoxic conditions in tissues, at levels where the endogenous NOS pathway becomes less active [31] leading to vasodilation and platelet inhibition. In addition to deoxyhemoglobin, nitrite can be converted to NO by NOS under hypoxic condition [32, 33]. Our study found an unexpected decrease of P-VASP^Ser239^ caused by L-NAME after addition of nitrite and deoxygenated erythrocytes. Therefore, total levels of P-VASP^Ser239^ appear, at least in part, dependent on interactions with NOS and raises the possibility of the presence of NOS enzymes in the platelets and/or the erythrocytes. These processes are in addition to the direct effects of erythrocytes in reducing nitrite to NO. Erythrocytes eNOS has been reported by several groups [34] as has platelet NOS [35] although the many reports on platelet NOS has been questioned by others [36]. It is also possible that the use of NOS inhibitors such as L-NAME are not specific and have effects on other aspects of the nitrite induced production of NO itself. Conversely, not all effects of NO on platelet function may be reflected in P-VASP^Ser239^ levels.

Thus, the total effects of the presence of erythrocytes on these processes appear to depend on the mechanism and kinetics of formation of NO and its release from the erythrocytes and transfer to platelets, processes we are studying further. Although the exact mechanism of how NO escapes erythrocytes is still not completely clear, NO release has been detected from interaction between nitrite and deoxygenated erythrocytes by several laboratories [8, 37]. In addition, NO-dependent vasodilation and platelet inhibition effects of nitrite reported by independent laboratories also confirm the production of NO from interaction of nitrite with erythrocytes [6, 9, 29]. Since NO produced inside erythrocytes by interaction between nitrite and deoxyhemoglobin is rapidly scavenged, nitrite has been proposed to react with a membrane metabolon such as the anion exchanger-1 (AE-1), carbonic anhydrase and deoxyhemoglobin [38]. The compartmentalization of NO might facilitate NO release. One recent study demonstrated that nitrite reacted hemoglobin such as metHb-NO and Hb-NO^+^ binds with the erythrocytic membrane at high affinity [39]. This nitrite reacted hemoglobin may compete with deoxy- and oxyhemoglobin for binding with AE-1 leading to facilitate NO release. In addition, it has been suggested that nitrite inhibits platelets through facilitating S-nitrosation of the erythrocytic membrane [9]. Such surface nitrosation of erythrocytes may facilitate NO release by transnitrosylation.

In conclusion, our results confirm that the interaction between nitrite with partially deoxygenated erythrocytes inhibits platelets by NO/sGC pathway. Further, these findings also confirm and extend our previous results suggesting that reduction of nitrite by partially deoxygenated erythrocytes in the circulation modulate platelet activation and blood clotting within the various parts of the vascular bed. They may also open the potential use of nitrite therapy to regulate thrombotic processes.

## Supporting information

S1 Fig L-NAME and ODQ inhibited P-VASP^Ser239^ induced by nitrite and deoxygenated erythrocytes. L-NAME (300 μM) or ODQ (10 μM) was incubated with PRP + erythrocytes (20% hematocrit) for 30 and 10 minutes, respectively and then samples were deoxygenated by helium. Nitrite (10 μM) was added to deoxygenated samples and incubated at 37 °C for 10 minutes. Data are mean ± SEM (n = 5). *P < 0.05 tested by one-way ANOVA with Tukey’s multiple comparison.
(TIF)

S2 Fig Representative Western blot bands of the effects of nitrite on P-VASP^Ser239^ in platelets in the presence of deoxygenated erythrocytes at different hematocrit values. PRP + erythrocytes (10, 15, 20, 30 and 40% hematocrit) were deoxygenated by helium. Nitrite (10 μM) was added to deoxygenated samples and incubated at 37 °C for 10 minutes.
(TIF)

S3 Fig Representative Western blot bands of the effects of nitrite and NO donors on P-VASP^Ser239^ in deoxygenated whole blood. Whole blood was deoxygenated by helium for 10 minutes. Nitrite (10 μM) was incubated in deoxygenated whole blood for 15 minutes while DEANONOate (1 μM) and sodium nitroprusside (10 μM) were incubated for 5 minutes, respectively.
(TIF)

## References

[1] Kim-Shapiro DB, Gladwin MT. Mechanisms of nitrite bioactivation. Nitric Oxide. 2014;38:58–68. doi: 10.1016/j.niox.2013.11.002 24315961 10.1016/j.niox.2013.11.002 PMC3999231

[2] Lundberg JO, Gladwin MT, Ahluwalia A, Benjamin N, Bryan NS, Butler A, et al Nitrate and nitrite in biology, nutrition and therapeutics. Nat Chem Biol. 2009;5:865–9. doi: 10.1038/nchembio.260 19915529 10.1038/nchembio.260 PMC4038383

[3] Srihirun S, Sriwantana T, Unchern S, Kittikool D, Noulsri E, Pattanapanyasat K, et al Platelet inhibition by nitrite is dependent on erythrocytes and deoxygenation. PLoS One. 2012;7:e30380 doi: 10.1371/journal.pone.0030380 22276188 10.1371/journal.pone.0030380 PMC3262819

[4] Akrawinthawong K, Park JW, Piknova B, Sibmooh N, Fucharoen S, Schechter AN. A flow cytometric analysis of the inhibition of platelet reactivity due to nitrite reduction by deoxygenated erythrocytes. PLoS One. 2014;9:e92435 doi: 10.1371/journal.pone.0092435 24642865 10.1371/journal.pone.0092435 PMC3958531

[5] Park JW, Piknova B, Huang PL, Noguchi CT, Schechter AN. Effect of blood nitrite and nitrate levels on murine platelet function. PLoS One. 2013;8:e55699 doi: 10.1371/journal.pone.0055699 23383344 10.1371/journal.pone.0055699 PMC3562242

[6] Velmurugan S, Kapil V, Ghosh SM, Davies S, McKnight A, Aboud Z, et al Antiplatelet effects of dietary nitrate in healthy volunteers: involvement of cGMP and influence of sex. Free Radic Biol Med. 2013;65:1521–32. doi: 10.1016/j.freeradbiomed.2013.06.031 23806384 10.1016/j.freeradbiomed.2013.06.031 PMC3878381

[7] Dautov RF, Stafford I, Liu S, Cullen H, Madhani M, Chirkov YY, et al Hypoxic potentiation of nitrite effects in human vessels and platelets. Nitric Oxide. 2014;40:36–44. doi: 10.1016/j.niox.2014.05.005 24858215 10.1016/j.niox.2014.05.005

[8] Liu C, Wajih N, Liu X, Basu S, Janes J, Marvel M, et al Mechanisms of human erythrocytic bioactivation of nitrite. J Biol Chem. 2015;290:1281–94. doi: 10.1074/jbc.M114.609222 25471374 10.1074/jbc.M114.609222 PMC4294492

[9] Wajih N, Liu X, Shetty P, Basu S, Wu H, Hogg N, et al The role of red blood cell S-nitrosation in nitrite bioactivation and its modulation by leucine and glucose. Redox Biol. 2016;8:415–21. doi: 10.1016/j.redox.2016.04.004 27156251 10.1016/j.redox.2016.04.004 PMC4864376

[10] Wajih N, Basu S, Jailwala A, Kim HW, Ostrowski D, Perlegas A, et al Potential therapeutic action of nitrite in sickle cell disease. Redox Biol. 2017;12:1026–39. doi: 10.1016/j.redox.2017.05.006 28511346 10.1016/j.redox.2017.05.006 PMC5430577

[11] Gambaryan S, Subramanian H, Kehrer L, Mindukshev I, Sudnitsyna J, Reiss C, et al Erythrocytes do not activate purified and platelet soluble guanylate cyclases even in conditions favourable for NO synthesis. Cell Commun Signal. 2016;14:16 doi: 10.1186/s12964-016-0139-9 27515066 10.1186/s12964-016-0139-9 PMC4982240

[12] Horstrup K, Jablonka B, Honig-Liedl P, Just M, Kochsiek K, Walter U. Phosphorylation of focal adhesion vasodilator-stimulated phosphoprotein at Ser157 in intact human platelets correlates with fibrinogen receptor inhibition. Eur J Biochem. 1994;225:21–7. 7925440 10.1111/j.1432-1033.1994.00021.x

[13] Massberg S, Gruner S, Konrad I, Garcia Arguinzonis MI, Eigenthaler M, Hemler K, et al Enhanced in vivo platelet adhesion in vasodilator-stimulated phosphoprotein (VASP)-deficient mice. Blood. 2004;103:136–42. doi: 10.1182/blood-2002-11-3417 12933589 10.1182/blood-2002-11-3417

[14] Burkhart JM, Vaudel M, Gambaryan S, Radau S, Walter U, Martens L, et al The first comprehensive and quantitative analysis of human platelet protein composition allows the comparative analysis of structural and functional pathways. Blood. 2012;120:e73–82. doi: 10.1182/blood-2012-04-416594 22869793 10.1182/blood-2012-04-416594

[15] Munzel T, Feil R, Mulsch A, Lohmann SM, Hofmann F, Walter U. Physiology and pathophysiology of vascular signaling controlled by guanosine 3’,5’-cyclic monophosphate-dependent protein kinase [corrected]. Circulation. 2003;108:2172–83. doi: 10.1161/01.CIR.0000094403.78467.C3 14597579 10.1161/01.CIR.0000094403.78467.C3

[16] Smolenski A, Bachmann C, Reinhard K, Honig-Liedl P, Jarchau T, Hoschuetzky H, et al Analysis and regulation of vasodilator-stimulated phosphoprotein serine 239 phosphorylation in vitro and in intact cells using a phosphospecific monoclonal antibody. J Biol Chem. 1998;273:20029–35. 9685341 10.1074/jbc.273.32.20029

[17] Parakaw T, Suknuntha K, Vivithanaporn P, Schlagenhauf A, Topanurak S, Fucharoen S, et al Platelet inhibition and increased phosphorylated vasodilator-stimulated phosphoprotein following sodium nitrite inhalation. Nitric Oxide. 2017;66:10–6. doi: 10.1016/j.niox.2017.02.008 28235634 10.1016/j.niox.2017.02.008

[18] Apostoli GL, Solomon A, Smallwood MJ, Winyard PG, Emerson M. Role of inorganic nitrate and nitrite in driving nitric oxide-cGMP-mediated inhibition of platelet aggregation in vitro and in vivo. J Thromb Haemost. 2014;12:1880–9. doi: 10.1111/jth.12711 25163536 10.1111/jth.12711

[19] Benz PM, Laban H, Zink J, Gunther L, Walter U, Gambaryan S, et al Vasodilator-Stimulated Phosphoprotein (VASP)-dependent and -independent pathways regulate thrombin-induced activation of Rap1b in platelets. Cell Commun Signal. 2016;14:21 doi: 10.1186/s12964-016-0144-z 27620165 10.1186/s12964-016-0144-z PMC5020514

[20] Park JW, Piknova B, Nghiem K, Lozier JN, Schechter AN. Inhibitory effect of nitrite on coagulation processes demonstrated by thrombelastography. Nitric Oxide. 2014;40:45–51. doi: 10.1016/j.niox.2014.05.006 24858214 10.1016/j.niox.2014.05.006 PMC4133308

[21] Megson IL, Sogo N, Mazzei FA, Butler AR, Walton JC, Webb DJ. Inhibition of human platelet aggregation by a novel S-nitrosothiol is abolished by haemoglobin and red blood cells in vitro: implications for anti-thrombotic therapy. Br J Pharmacol. 2000;131:1391–8. doi: 10.1038/sj.bjp.0703731 11090112 10.1038/sj.bjp.0703731 PMC1572482

[22] Wennmalm A, Benthin G, Petersson AS. Dependence of the metabolism of nitric oxide (NO) in healthy human whole blood on the oxygenation of its red cell haemoglobin. Br J Pharmacol. 1992;106:507–8. 1504736 10.1111/j.1476-5381.1992.tb14365.x PMC1907547

[23] Doyle MP, Pickering RA, DeWeert TM, Hoekstra JW, Pater D. Kinetics and mechanism of the oxidation of human deoxyhemoglobin by nitrites. J Biol Chem. 1981;256:12393–8. 7298665

[24] Anfossi G, Russo I, Massucco P, Mattiello L, Balbo A, Cavalot F, et al Studies on inhibition of human platelet function by sodium nitroprusside. Kinetic evaluation of the effect on aggregation and cyclic nucleotide content. Thromb Res. 2001;102:319–30. 11369425 10.1016/s0049-3848(01)00240-7

[25] Feelisch M. The use of nitric oxide donors in pharmacological studies. Naunyn Schmiedebergs Arch Pharmacol. 1998;358:113–22. 9721012 10.1007/pl00005231

[26] Grossi L, D’Angelo S. Sodium nitroprusside: mechanism of NO release mediated by sulfhydryl-containing molecules. J Med Chem. 2005;48:2622–6. doi: 10.1021/jm049857n 15801852 10.1021/jm049857n

[27] Rochelle LG, Kruszyna H, Kruszyna R, Barchowsky A, Wilcox DE, Smith RP. Bioactivation of nitroprusside by porcine endothelial cells. Toxicol Appl Pharmacol. 1994;128:123–8. doi: 10.1006/taap.1994.1189 8079345 10.1006/taap.1994.1189

[28] Huang Z, Shiva S, Kim-Shapiro DB, Patel RP, Ringwood LA, Irby CE, et al Enzymatic function of hemoglobin as a nitrite reductase that produces NO under allosteric control. J Clin Invest. 2005;115:2099–107. doi: 10.1172/JCI24650 16041407 10.1172/JCI24650 PMC1177999

[29] Crawford JH, Isbell TS, Huang Z, Shiva S, Chacko BK, Schechter AN, et al Hypoxia, red blood cells, and nitrite regulate NO-dependent hypoxic vasodilation. Blood. 2006;107:566–74. doi: 10.1182/blood-2005-07-2668 16195332 10.1182/blood-2005-07-2668 PMC1895612

[30] Carreau A, El Hafny-Rahbi B, Matejuk A, Grillon C, Kieda C. Why is the partial oxygen pressure of human tissues a crucial parameter? Small molecules and hypoxia. J Cell Mol Med. 2011;15:1239–53. doi: 10.1111/j.1582-4934.2011.01258.x 21251211 10.1111/j.1582-4934.2011.01258.x PMC4373326

[31] Ostergaard L, Stankevicius E, Andersen MR, Eskildsen-Helmond Y, Ledet T, Mulvany MJ, et al Diminished NO release in chronic hypoxic human endothelial cells. Am J Physiol Heart Circ Physiol. 2007;293:H2894–903. doi: 10.1152/ajpheart.01230.2006 17720765 10.1152/ajpheart.01230.2006

[32] Gautier C, van Faassen E, Mikula I, Martasek P, Slama-Schwok A. Endothelial nitric oxide synthase reduces nitrite anions to NO under anoxia. Biochem Biophys Res Commun. 2006;341:816–21. doi: 10.1016/j.bbrc.2006.01.031 16442076 10.1016/j.bbrc.2006.01.031

[33] Webb AJ, Milsom AB, Rathod KS, Chu WL, Qureshi S, Lovell MJ, et al Mechanisms underlying erythrocyte and endothelial nitrite reduction to nitric oxide in hypoxia: role for xanthine oxidoreductase and endothelial nitric oxide synthase. Circ Res. 2008;103:957–64. doi: 10.1161/CIRCRESAHA.108.175810 18818408 10.1161/CIRCRESAHA.108.175810 PMC2841343

[34] Porro B, Eligini S, Squellerio I, Tremoli E, Cavalca V. The red blood cell: a new key player in cardiovascular homoeostasis? Focus on the nitric oxide pathway. Biochem Soc Trans. 2014;42:996–1000. doi: 10.1042/BST20140122 25109992 10.1042/BST20140122

[35] Shah A, Passacquale G, Gkaliagkousi E, Ritter J, Ferro A. Platelet nitric oxide signalling in heart failure: role of oxidative stress. Cardiovasc Res. 2011;91:625–31. doi: 10.1093/cvr/cvr115 21502370 10.1093/cvr/cvr115

[36] Bohmer A, Gambaryan S, Tsikas D. Human blood platelets lack nitric oxide synthase activity. Platelets. 2015;26:583–8. doi: 10.3109/09537104.2014.974024 25360996 10.3109/09537104.2014.974024

[37] Fens MH, Larkin SK, Oronsky B, Scicinski J, Morris CR, Kuypers FA. The capacity of red blood cells to reduce nitrite determines nitric oxide generation under hypoxic conditions. PLoS One. 2014;9:e101626 doi: 10.1371/journal.pone.0101626 25007272 10.1371/journal.pone.0101626 PMC4090171

[38] Gladwin MT, Crawford JH, Patel RP. The biochemistry of nitric oxide, nitrite, and hemoglobin: role in blood flow regulation. Free Radic Biol Med. 2004;36:707–17. doi: 10.1016/j.freeradbiomed.2003.11.032 14990351 10.1016/j.freeradbiomed.2003.11.032

[39] Salgado MT, Cao Z, Nagababu E, Mohanty JG, Rifkind JM. Red blood cell membrane-facilitated release of nitrite-derived nitric oxide bioactivity. Biochemistry. 2015;54:6712–23. doi: 10.1021/acs.biochem.5b00643 26478948 10.1021/acs.biochem.5b00643

