# Supplementary figures and images for: Phosphorylated vasodilator-stimulated phosphoprotein (P-VASPSer239) in platelets is increased by nitrite and partially deoxygenated erythrocytes
Source: PLoS One. 2018 Mar 5;13(3):e0193747. doi: 10.1371/journal.pone.0193747 (PMC5837118; doi:10.1371/journal.pone.0193747)

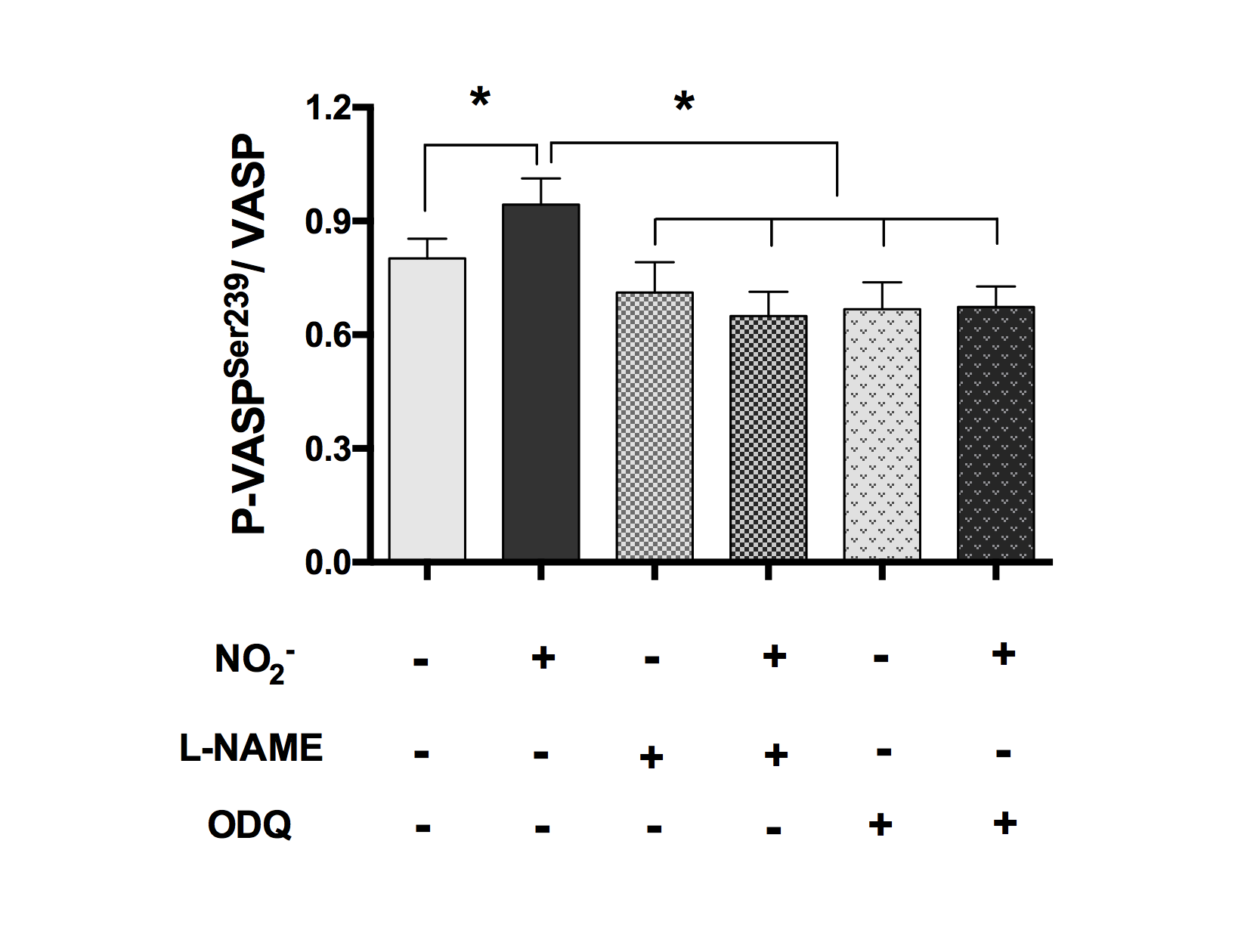

Supplement: S1 Fig — L-NAME (300 μM) or ODQ (10 μM) was incubated with PRP + erythrocytes (20% hematocrit) for 30 and 10 minutes, respectively and then samples were deoxygenated by helium. Nitrite (10 μM) was added to deoxygenated samples and incubated at 37 °C for 10 minutes. Data are mean ± SEM (n = 5). *P < 0.05 tested by one-way ANOVA with Tukey’s multiple comparison. (TIF) [file pone.0193747.s001.tif]

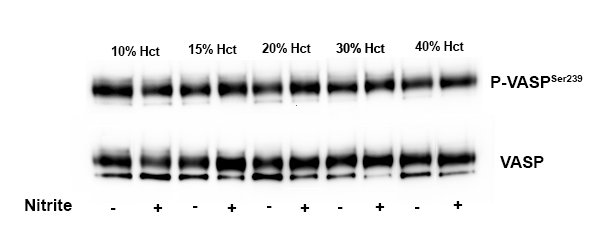

Supplement: S2 Fig — PRP + erythrocytes (10, 15, 20, 30 and 40% hematocrit) were deoxygenated by helium. Nitrite (10 μM) was added to deoxygenated samples and incubated at 37 °C for 10 minutes. (TIF) [file pone.0193747.s002.tif]

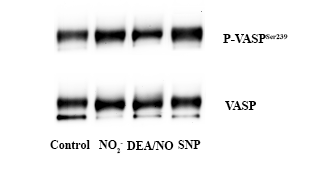

Supplement: S3 Fig — Whole blood was deoxygenated by helium for 10 minutes. Nitrite (10 μM) was incubated in deoxygenated whole blood for 15 minutes while DEANONOate (1 μM) and sodium nitroprusside (10 μM) were incubated for 5 minutes, respectively. (TIF) [file pone.0193747.s003.tif]
